# Parallel evolution of passive and active defence in land snails

**DOI:** 10.1038/srep35600

**Published:** 2016-11-11

**Authors:** Yuta Morii, Larisa Prozorova, Satoshi Chiba

**Affiliations:** 1 Forest Ecosystem Management Group, Department of forest Science, Graduate School of Agriculture, Hokkaido University, Sapporo 0608589, Japan; 2 Graduate School of Life Sciences & Center for Northeast Asian Studies, Tohoku University, Sendai 9808576, Japan; 3 Institute of Biology and Soil Science, Far East Branch, Russian Academy of Sciences, Vladivostok 690022, Russia

## Abstract

Predator-prey interactions are major processes promoting phenotypic evolution. However, it remains unclear how predation causes morphological and behavioural diversity in prey species and how it might lead to speciation. Here, we show that substantial divergence in the phenotypic traits of prey species has occurred among closely related land snails as a result of adaptation to predator attacks. This caused the divergence of defensive strategies into two alternatives: passive defence and active defence. Phenotypic traits of the subarctic *Karaftohelix* land snail have undergone radiation in northeast Asia, and distinctive morphotypes generally coexist in the same regions. In these land snails, we documented two alternative defence behaviours against predation by malacophagous beetles. Furthermore, the behaviours are potentially associated with differences in shell morphology. In addition, molecular phylogenetic analyses indicated that these alternative strategies against predation arose independently on the islands and on the continent suggesting that anti-predator adaptation is a major cause of phenotypic diversity in these snails. Finally, we suggest the potential speciation of *Karaftohelix* snails as a result of the divergence of defensive strategies into passive and active behaviours and the possibility of species radiation due to anti-predatory adaptations.

How does phenotypic divergence and radiation occur in nature? This has been a major question of evolutionary biology since Darwin’s work with finches^1^,^2^,^3^,^4^,^5^,^6^. Phenotypic radiation can be classified into two main categories—ecological radiation (i.e., adaptive)^3^ and non-ecological radiation (i.e., non-adaptive)^3^—although the mechanisms and patterns underlying these categories are still not fully understood^4^,^6^,^7^. According to Schluter^4^, ecological radiation is the evolution of ecological and phenotypic diversity within a rapidly multiplying lineage. Cases of radiation that do not show a clear correlation between phenotypic divergence and environmental interactions are considered to be non-ecological^2^,^4^. Many examples of ecological radiation have been demonstrated^4^,^8^,^9^,^10^,^11^,^12^,^13^,^14^,^15^,^16^,^17^,^18^, although the mechanisms of this radiation still remain unclear^4^,^6^,^7^.

The “ecological theory” of ecological radiation has been the major synthesis of ideas that explains the processes driving the ecological divergence of lineages^1^,^4^,^19^,^20^,^21^,^22^. In this theory, ecological radiation is ultimately the outcome of divergent natural selection stemming from environmental pressures and resource competition. In other words, this theory essentially follows the ideas behind the concept of niches^3^,^4^,^23^.

However, predator-prey interactions can be a major selective force along with resource competition in affecting morphological and behavioural traits^24^, habitat use^25^ and speciation^26^. Based on fossil records, Vermeij^27^ suggested that coevolution between prey and predators is a significant cause of the evolution in phenotypic diversity and radiation. However, it remains unclear how divergence of the phenotypes and speciation of prey or predators occurs under specific predator-prey interactions.

Land snails are an excellent system to investigate phenotypic evolution because of their high shell shape and colour variability, low mobility and strict habitat requirements^26^,^28^,^29^,^30^,^31^,^32^,^33^. Indeed, a number of examples of both ecological^29^,^34^ and non-ecological radiation^35^,^36^ have been reported in land snails.

Here, we focused on the subarctic land snails of the genus *Karaftohelix* in northeast Asia. Specifically, we examined snails on two northeastern islands in the Japanese Archipelago (Hokkaido and Honshu islands) and in southern far-east Russia (Figs 1 and 2). This group of land snails provides an excellent system to investigate phenotypic divergence via speciation because these snails have many divergent phenotypic traits (shell colour and pattern, shell surface sculpture including periostracal hairs or scales, shell shape and size, and behaviour) that differ in their levels of inter- and intra-species variation, and these variations usually indicate a sympatric distribution pattern^37^,^38^. Two of these snail species—*Karaftohelix* (*Ainohelix*) *editha* and *Karaftohelix* (*Ezohelix*) *gainesi*—on Hokkaido Island illustrate the extreme inter- and intra-specific levels of phenotypic variation of this group^38^. *K. editha* and *K. gainesi* have very different shell morphologies; therefore, these species previously belonged to different genera. However, these two species are current nearly indistinguishable both genetically and anatomically^38^. Because frequent hybridization has occurred between these two species, it is likely that divergence of the phenotypic traits of *K. editha* and *K. gainesi* evolved relatively rapidly due to natural selection^38^.

However, the factors driving this natural selection are not clear because *K. editha* and *K. gainesi* often inhabit the same region without divergence in habitat use^38^. Therefore, differences in habitat may not be the main selective pressure^38^. On the other hand, it is plausible that the phenotypic differences between land snails in this region have evolved in part due to predation pressure because predators that specialize on land snails, including some types of carabid beetles, are distributed throughout northeastern Asia^39^. Some species of carabid beetles were well known as the specialist predators of land snails^40^,^41^. For example, *Damaster blaptoides* and *Acoptolabrus gehinii* are distributed throughout most of Hokkaido Island as are *K. editha* and *K. gainesi*. It is likely that these carabid beetles prey on *K. editha* and *K. gainesi* because there are a few other large snail species on Hokkaido, but no species other than *K. editha* and *K. gainesi* are distributed throughout all of Hokkaido^42^.

Although a number of land snail predators are known^40^,^41^, there are not many malacophagous species on Hokkaido and southern far-east Russia. In addition, all of the malacophagous species other than carabid beetles are rare in these regions. Mammals and birds can also be predators of these land snails, but there is no evidence of such predation except by a chipmunk, *Tamias sibiricus*, in these areas. However, *T. siviricus* is omnivorous, and they prey on relatively low numbers of snails^43^; therefore, this species may not be an important snail predator.

Malacophagous flat worms and a burying beetle, *Phosphuga atrata*, were also distributed in these regions but also relatively rare. In contrast, carabid beetles, especially *D. blaptoides* and *A. gehinii*, are very abundant on Hokkaido, and *Coptolabrus smaragdinus* and *Acoptolabrus schrencki* on southern far-east Russia. Thus, it is likely that the phenotypic divergence and speciation of land snails in northeast Asia have been driven by predation pressure from carabid beetles.

To test this hypothesis, we examined the morphology of several land snail species in northeastern Asia, exposed them to natural predators, and observed their defensive responses. Second, we estimated the evolutionary relationships among the different species and morphotypes using molecular phylogenetic approaches. Finally, we discussed the distinctive defence behaviours that *Karaftohelix* snails display and how these behaviour and defence-related morphologies have evolved and resulted in species radiation.

## Materials and Methods

### Samples

**Figure 1:**
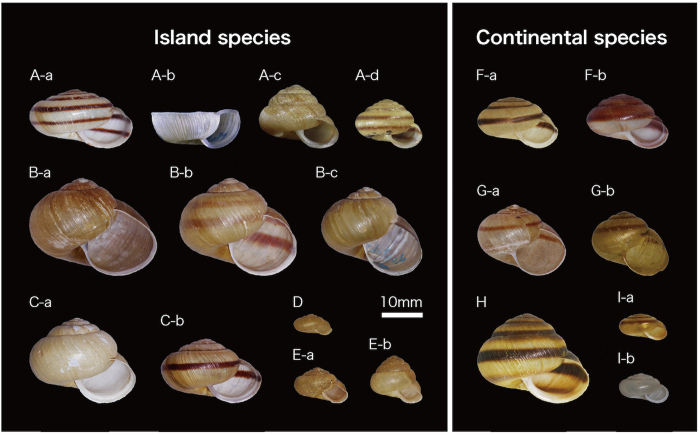
Species of land snails from the genus *Karaftohelix* in northeast Asia. (**A**) *Karaftohelix* (*Ainohelix*) *editha*, -a. from Horokanai (site no. 11 in Supplementary Table 1 and Fig. 2, Hokkaido), -b. from Urakawa (no. 28, Hokkaido), -c. from Bibai (no. 19-1, Hokkaido), -d. from Kitami (no. 12, Hokkaido); (**B**) *Karaftohelix* (*Ezohelix*) *gainesi*, -a. from Bibai (no. 19-1, Hokkaido), -b. from Sapporo (no. 24, Hokkaido), -c. from Yagishiri (no. 9, Hokkaido); (**C**) *Karaftohelix blakeana*, -a. from Soya (no. 4, Hokkaido), -b. from Rebun (no. 5-1, Hokkaido); (**D**) *Karaftohelix* (*Paraegista*) *apoiensis* from Samani (no. 30-1, Hokkaido); (**E**) *Karaftohelix* (*Paraegista*) *takahidei*, -a. from Sapporo (no. 24, Hokkaido), -b. from Shakotan (no. 20, Hokkaido); (**F**)-a,b. *Karaftohelix middendorffi* from Bikin (no. 2, Russia); (**G**) *Karaftohelix selskii*, -a. from Bikin (no. 2, Russia), -b. from Krasny Yar village (no. 1, Russia); (**H**) *Karaftohelix maackii* from Bikin (no. 2, Russia); and (**I**) *Karaftohelix ussuriensis*, -a. from Krasny Yar village (no. 1, Russia), -b. from Russky Island (no. 3, Russia).

**Figure 2:**
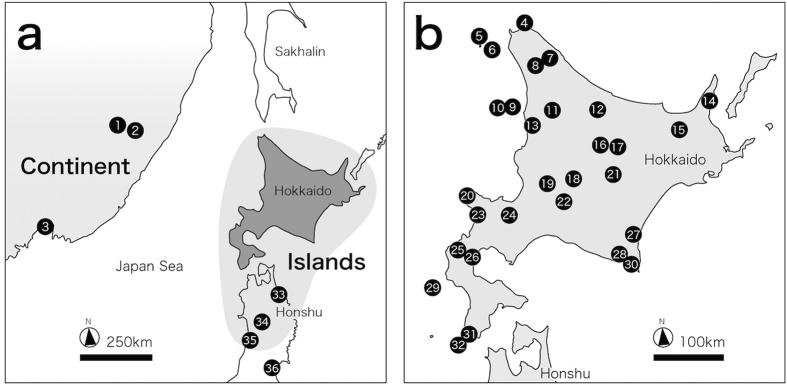
A map of the sampling localities of the snails used in this study. The numerals correspond to the locality numbers in Supplementary Table 1. The maps were created using the software “Adobe Illustrator, [CS5, Macintosh version], ( https://www.adobe.com/jp/support/downloads/ilmac.html)” and “Map data: Google, DigitalGlobe”.

Nine genetically related bradybaenid species were collected and analysed. Five species of *Karaftohelix*, *K.* (*Ainohelix*) *editha*, *K.* (*Ezohelix*) *gainesi*, *K. blakeana*, *K*. (*Paraegista*) *apoiensis* and *K*. (*P.*) *takahidei*, endemic to Japan (mainly on Hokkaido; Fig. 1; Supplementary Table 1)^42^,^44^ were collected. Four continental species of the genus *Karaftohelix*, *K. maackii*, *K. middendorffi*, *K. selskii* and *K. ussuriensis* (Fig. 1) were also collected from three populations of two regions in southern far-east Russia (Fig. 2; Supplementary Table 1). All bradybaenid land snails used in this study are large species (larger than 8 mm). We found that several congeneric species coexisted in locations on Hokkaido Island and in far-east Russia. *Acusta despecta* (Pulmonata: Bradybaenidae) was used as an outgroup for phylogenetic analyses because it has been shown that the genus *Acusta* is the sister genus of *Karaftohelix*^45^,^46^.

### Behavioural observations

In total, 55 adult snails from eight species (four island species and four continental species; Supplementary Table 1) were used for behavioural observations. The foot of each snail was given an artificial external stimulus by pushing on it with fine-tip tweezers, and the response behaviour of the snail was observed. All snails used for behavioural observations were cultured individually in plastic cases (15.5 cm × 11.0 cm × 4.5 cm) with wet tissue paper at the bottom in room temperature (20~25 °C) for three to seven days before observations. Some trials were recorded with a video camera (HDR-XR500 V; SONY, Japan).

In addition, ten adult *K. editha* and *K. gainesi* snails from Bibai (locality no. 19-3 in Fig. 2 and Supplementary Table 1) were placed with two species of malacophagous carabid beetles, *Damaster blaptoides* and *Acoptolabrus gehinii*, collected primarily from Bibai (one beetle was from Furano, no. 18; Fig. 2; Supplementary Table 1). In each experiment, one beetle and one snail were put into a plastic case (15.5 cm × 11.0 cm × 4.5 cm) with horticultural soil at the bottom for 15 minutes under low-intensity light, and the behaviour of the snail in avoiding predatory attacks by the beetles was observed. All beetles were fed sufficient amounts of fish meat sausage three days before trials. Some trials were recorded with a video camera (HDR-XR500 V; SONY, Japan).

### Morphological analyses

**Figure 3:**
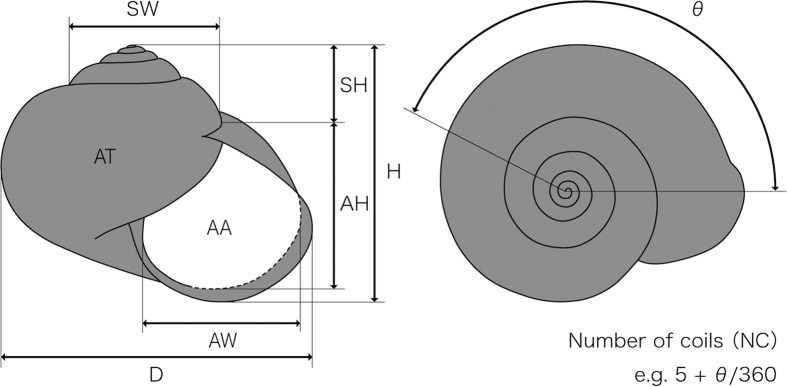
Characteristics measured for the morphological analysis of the shell. AH, aperture height; AW, aperture width; D, shell diameter; H, total shell height; SH, shell height above the aperture; SW, spire width; AA, aperture area; AT, total area including AA; NC, Number of coils.

Shell morphological analyses were conducted for 165 individuals of nine species (Supplementary Table 1). Nine shell morphological traits were measured from pictures of the shell (Fig. 3). Traits included: aperture height (AH), aperture width (AW), shell diameter (D), total shell height (H), shell height above the aperture (SH), spire width (SW), number of coils (NC), aperture area (AA) and total area in the shell (AT). The shell shape and size were analysed separately. A principal component analysis (PCA) was used for the analysis of shell shape; this was conducted with JMP software (SAS Institute, North Carolina) using mean values of the five lengths relative to the shell diameter (AH/D, AW/D, H/D, SH/D, SW/D and NC/D) from each species and from each locality. In addition, the mean relative aperture area (AA/AT) of each species from each locality was compared among the different behavioural traits. The shell diameter (D) of each species was also compared among the different behavioural traits for the size analysis. All measurements are shown in Supplementary Table 2.

### Molecular methods

Foot tissue was homogenized in 300 μL cetyltrimethylammonium bromide (CTAB) solution [2% CTAB (w/v), 100 mM Tris (pH 8.0), 20 mM EDTA (pH 8.0), 1.4 M NaCl] and 20 μL of 10 mg/mL proteinase K, incubated at 60 °C for approximately 1 hour, extracted once with phenol/chloroform and precipitated with two volumes of ethanol. The DNA pellet was then rinsed with 70% ethanol, vacuum-dried for approximately 1 hour and dissolved in 50 μL of distilled water.

To estimate the phylogenetic relationships among the collected snails, sequenced fragments were sampled from two mitochondrial DNA regions (cytochrome oxidase subunit 1 (CO1) gene (~530 bp) and 16S ribosomal DNA (16S; ~700 bp)) and from two nuclear DNA regions (ribosomal internal transcribed spacer regions 1 and 2 (ITS; ~1200 bp) and external transcribed spacer region (ETS; ~380 bp)). Polymerase chain reaction (PCR) conditions and the primers used are shown in Supplementary Table 3. The PCR products were purified using Exo-SAP-IT (Amersham Biosciences, Little Chalfont, Buckinghamshire, UK). The sequencing cycle was carried out with both forward and reverse primers using ~80–100 ng of PCR product in the reaction and the BigDye^TM^ Terminator v3.0 Cycle Sequencing Ready Reaction Kit (Applied Biosystems, California). The DNA sequences were electrophoresed on a 310 Genetic Analyser or a 3130 Genetic Analyser (both Applied Biosystems, California).

### Phylogenetic analyses

Sequences were aligned using MUSCLE v3.8^47^, and the results were cleaned of problematic alignment blocks using GBLOCKS v0.91^48^ with the default parameters. Gene trees were constructed using Bayesian inference (BI), maximum likelihood (ML), maximum parsimony (MP) and neighbour joining (NJ) models based on the combined dataset with all sequences (16S, CO1, ITS and ETS).

Prior to the ML and BI analyses, the appropriate models of sequence evolution were selected with Kakusan software version 4–4.0.2011.05.28 49,50, and all the sequences were combined. BI analysis used MrBayes v3.1.2^51^. Tree spaces were explored using two concurrent runs with four simultaneous Markov chain Monte Carlo (MCMC) simulations for 10 million generations with sampling every 100 generations for the combined data set of all sequences (16S, CO1, ITS and ETS). The number of generations before stationarity of likelihood values was obtained and was estimated with TRACER v1.5 software^52^ such that the effective sample sizes of all parameters were more than 190 after the burn-in. The heating parameters were set to 0.15. After discarding the first 10001 trees as the burn-in, the 50% majority rule consensus tree and the posterior probabilities of nodes in the tree were obtained.

The ML analysis was performed with TREEFINDER v2008 53 under the maximum likelihood criterion. The MP and NJ trees were reconstructed using MEGA v6.0^54^. Prior to the MP and NJ analyses, the 16S, CO1, ITS, and ETS sequences were combined using MEGA. Nodal support for the ML, MP and NJ analyses were assessed using bootstrap analyses^55^ with 1000 replications.

## Results

### Behavioural observations

**Figure 4:**
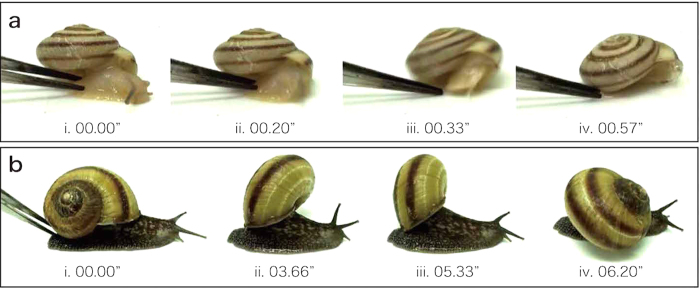
The sequences of response behaviour to external stimulus of two genetically related species, *Karaftohelix editha* and *Karaftohelix gainesi*. The numbers under each picture indicate elapsed time (seconds) from the applied stimulus. (**a**) i–vi. The behaviour of *K. editha* from Wakkanai (no. 4, Hokkaido). *K. editha* pulled their soft body into their shell. (**b**) i–vi. The behaviour of *K. gainesi* from Sapporo (no. 24, Hokkaido). *K. gainesi* shows a unique behaviour by swinging their shell around instead of pulling their body into their shell. The time from the start of the swing (iii) to the end (vi) was less than one second.

The observed behaviours were classified into two main categories—passive defence and active defence. Almost all individuals from all six species (*K. editha*, *K. blakeana*, *K. takahidei*, *K. maackii*, *K. middendorffi* and *K. ussuriensis*) retracted their soft body into their shells very quickly, which is a passive defence behaviour (Table 1; Fig. 4a; Supplementary Movie 1, 3, 4, 5). In contrast, no individuals from the other two species (*K. gainesi* and *K. selskii*) retracted their soft body into the shell. Rather, they became even more active than before the external stimulus and vigorously swung their shells. This motion was usually repeated several times at the same frequency (approximately one swing every three seconds; Table 1; Fig. 4b; Supplementary Movie 2, 6). Two individuals of *K. blakeana* did not show a quick response and finally retracted their soft body into the shell, and one individual of *K. gainesi* and two individuals of *K. selskii* showed a different behaviour in which they created bubbles around their soft body (indicated as “other behaviours” in Table 1; Supplementary Movie 7). The behaviour of *K. editha*, *K. blakeana* and *K. gainesi* was also observed tentatively in the wild, and the same behaviour observed in the laboratory was seen in the wild.

These extremely different behaviours were clearly associated with snail species. All species were separated into two groups: “passive defence species” (*K. editha*, *K. blakeana*, *K. takahidei*, *K. maackii*, *K. middendorffi* and *K. ussuriensis*) and “active defence species” (*K. gainesi* and *K. selskii*). When the snails received external stimulus, the passive defence species retracted their soft body into the shell, and the active defence species showed aggressive behaviours.

These behaviours were also observed when the malacophagous carabid beetles attacked the snails (n = 10 for each of two snail species, *K. editha* and *K. gainesi*; Supplementary Table 1). The *K. editha* eventually escaped from the predator by retracting deep inside the shell (Supplementary Movie 8). In contrast, *K. gainesi* escaped from the predator by flipping away or even knocking the predator over with its shell (Supplementary Movie 9, 10).

### Morphological analyses

**Figure 5:**
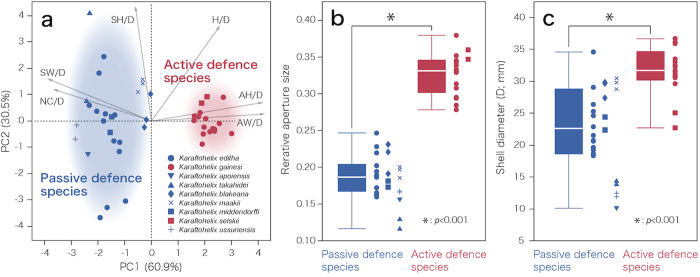
The results of morphological analyses. (**a**) Scatter plots of the principal component scores for shell morphologies. Passive and active defence species were clearly separated; red and blue coloured clusters indicate passive and active defence species, respectively. (**b**) Box plots of relative aperture size for passive and active defence species. (**c**) Box plots of shell diameter for passive and active defence species.

More than 91% of the variation in shell morphology among the individual snails was explained by two principal components (PC1 and PC2; Table 2; Fig. 5a). The AW/D, AH/D, SW/D and NC/D had large loading values (more than 0.8 or less than −0.8). These four traits are related to the relative aperture size; thus, PC1 can be interpreted as a factor explaining the relative aperture size. Two other factors, H/D and SH/D, had high loading values on PC2 (more than 0.8). Therefore, PC2 can be interpreted as a factor explaining the relative shell height.

Morphologically, passive and active defence species were clearly separated from each other based on PC1 scores (Steel-Dwass test, *P* < 0.001). In contrast, PC2 scores were not significantly different between the behavioural groups (Steel-Dwass test, *P* > 0.05). These results clearly indicated that the relative aperture size was much larger in active defence species than in passive defence species. The PCA results were confirmed more directly by comparing the relative aperture area (AA/AT) between passive and active defence species (Fig. 5b; Steel-Dwass test, *P* < 0.001).

In addition, the shell diameter (D) was significantly larger in active defence species than in passive defence species (Fig. 5c; Steel-Dwass test, *P* < 0.001). This might indicate that the two different defensive behaviours were associated with different shell sizes, although the shell size of *K. selskii* was barely larger than the passive defence species.

### Phylogenetic analyses

**Figure 6:**
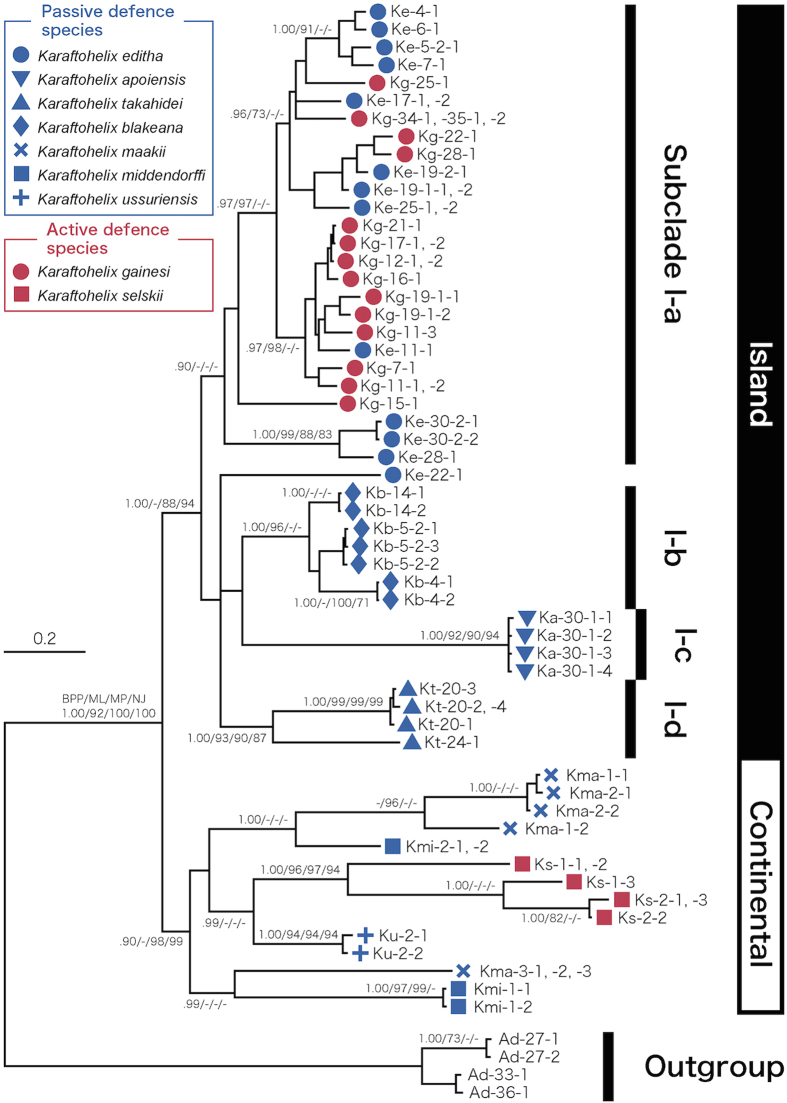
The Bayesian phylogenetic tree inferred from a combined data set of nDNA and mtDNA sequences (16S, CO1, ITS and ETS; approximately 2600 bp). Numbers on each branch represent the Bayesian posterior probability (BPP; values <0.90 are not shown), and the bootstrap support for clades resolved in the ML, MP and NJ analyses (ML, MP and NJ; values <70% are not shown).

In the molecular phylogenetic analyses, 74 individuals of the nine species as well as the outgroup taxa were analysed, and 60 haplotypes were detected. All analyses (BI, ML, MP and NJ) resulted in nearly identical topologies. The inferred phylogenetic relationships among the haplotypes are shown in Fig. 6.

Two major clades were identified, and these clades corresponded to island and continental species. The monophyly of the island clade was strongly supported (Bayesian posterior probability (BPP) = 1.00, bootstrap support value (BV) for ML, MP and NJ analyses = 65, 88 and 94%, respectively). The monophyly of the continental clade was also well supported in all trees except for the ML tree (BPP = 0.90, BV for MP and NJ = 98 and 99%, respectively). In the ML analyses, the relationships among the continental species and populations were unclear.

The island clade was further subdivided into approximately four groups (subclades I-a, I-b, I-c and I-d; Fig. 6). Each subclade was represented by only a single species except for subclade I-a, which included *K. editha* and *K. gainesi*. Only one individual of *K. editha* from Yubari (Ke-22–1) was not included in subclade I-a.

In the continental clade, individuals collected from the different regions tended to have very distinctive genotypes even within the same species (e.g., *K. maackii* and *K. middendorffi*). However, obvious subclades were not recognized, and the relationships among haplotypes were not clearly resolved due to incongruence of the topologies among the BI, ML, MP and NJ models.

The passive and active defence species were both separated into islands and continental clades indicating that the divergence of passive and active defence species occurred independently on the island and on the continent (Fig. 6).

## Discussion

Two alternative anti-predator behaviours, passive and active defence, were documented among closely related *Karaftohelix* snails. Although further studies are needed, the results of feeding experiments suggest that these two alternative behaviours have the same function—avoiding predation by malacophagous carabid beetles. The passive defence snails use their shell as a “shield” to defend their soft body from the predator’s attack, whereas the active defence snails use their shell as a “club” to hit the predators and knock them over. This study is one of only a few to report on land snails using their shell for active defence by swinging it against a predator. One example of a similar but more obscure behaviour in a Japanese bradybaenid land snail, *Acusta despecta*, has been described when these snails are attacked by the larvae of fireflies^56^,^57^,^58^.

The hypothesis that predation pressure led to morphological divergence in these snails seems to be reasonable because the alternative behaviours demonstrated by the different taxa are associated with differences in shell shape and size. The shell morphology analyses indicated that the relative aperture size of the shell was strongly associated with behavioural differences (Fig. 5a,b). In addition, the shell diameter might be larger in the active defence species than in the passive defence species when coexisting species are compared (Fig. 5c). For the passive defence snails, which retract as an anti-predatory behaviour, a shell with a narrower relative aperture prevents the predator from inserting its head in the shell^41^,^57^,^58^. The outer lip of the adult shell of all the passive defence species is also markedly thickened, suggesting that this characteristic is effective in protecting the shell from being broken by the predator when the soft body is deeply retracted into the shell^41^,^57^,^58^.

In contrast, active defence snails swing their shell as an anti-predatory behaviour, and a larger relative aperture might allow development of strong muscle to swing the shell around the soft body. In addition, a large relative aperture size relates to a relatively large body size—this can help shake off the predator and can even damage the predator. Aperture size is positively correlated with foot size^59^ and thus with muscle mass. Thus, these two different defensive strategies are incompatible because there is a fundamental functional trade-off between those that use the shell as a “shield” versus a “club”. The morphological analyses support this idea because there were no intermediate morphotypes between species with both passive and active defence strategies.

The phylogenetic analyses clearly suggested that the passive and active defence species and the morphotypes related to these defensive strategies arose independently on the islands and the continent, although the divergence pattern of the island clade was more complex than continental clades (Fig. 6). This may suggest that the divergence of passive and active defence strategies and island speciation has been ongoing. This pattern—parallel evolution of similar adaptive traits in several independent regions—strongly implicates natural selection against predation pressure as the cause of the evolution of these traits^4^,^5^,^6^,^7^,^12^. It is unlikely that the morphological differences among the several species are due to major differences in habitat because there were no obvious differences in the local microhabitats occupied by species when they coexisted. The hypothesis that predation pressure led to speciation is a uncommon explanation of morphological divergence because adaptation to different microhabitats is a major factor underlying phenotypic divergence among species^4^,^6^,^8^,^14^,^15^,^60^.

The divergence in body size between coexisting passive and active defence species may promote the evolution of reproductive isolation between these species—especially between *K. editha* and *K. gainesi* on Hokkaido Island. Although the shell diameter of *K. selskii* is only slightly larger than the sympatric *K. middendorffi*, the size of the soft body is larger in the former than the latter because of the larger relative aperture size of the former. In bradybaenid snails, differences in body size cause reproductive isolation because of the presence of size-assortative mating^61^. Although further experimental approaches are needed, this study implies that divergence in defence strategies against the same predator can cause speciation in *Karaftohelix*.

The patterns of *Karaftohelix* diversification indicates that an ecological radiation occurred among these land snails because they clearly share a common ancestry (Fig. 6)^45^,^46^. Although the exact ages of speciation of each species are difficult to estimate, divergence of passive and active defence species appears to have started 1–3 Ma within the islands and continent based on the evolutionary rate of bradybaenid 16SrRNA^62^. This suggests that there has been enough time for the populations to diverge into highly distinctive phenotypes.

The active defence species *K. gainesi* coexists with one or two congeneric passive defence species on the islands, and another active defence species *K. selskii* coexists with two or three congeneric passive defence species on the continent. However, no clear relationship is found between morphology and number of coexisting congeneric species. A large overlap of habitat and/or resource use among these snail species suggests that interspecific competition among sympatric species is weak. Differences in shell size among the sympatric passive defence species are unlikely to be caused by interspecific competition because of no difference in habitat use among these species. Although further analyses are needed, we speculate that the larger passive defence species is more advantageous to protect the shell from being broken by the beetle and the smaller passive defence species is more advantageous to prevent the beetle from inserting its head in the shell.

Therefore, predation is shaping the evolutionary change among these land snails. Morphological changes in relative aperture size represent an ecological trade-off and only one strategy can be employed.

This type of radiation does not follow the existing ecological models of radiation. The evolutionary pattern of the bradybaenid land snails observed here seems to follow the model of prey species divergence by “apparent competition”^63^. Similar examples of phenotypic divergence of prey driven by a small number of predator species have been shown in some previous studies on freshwater and land snails^64^,^65^. Although further studies are needed to clarify the genetic patterns of speciation, morphological variations and behavioural traits in these snail, the present findings shed light on ecological factors other than resource competition that are important forces to drive phenotypic divergence and species radiation.

## Additional Information

**How to cite this article**: Morii, Y. *et al*. Parallel evolution of passive and active defence in land snails. *Sci. Rep.* **6**, 35600; doi: 10.1038/srep35600 (2016).

**Publisher’s note:** Springer Nature remains neutral with regard to jurisdictional claims in published maps and institutional affiliations.

## Supplementary Material

Supplementary Information

Supplementary Video

Supplementary Video

Supplementary Video

Supplementary Video

Supplementary Video

Supplementary Video

Supplementary Video

Supplementary Video

Supplementary Video

Supplementary Video

Table s1

Table s2

Table s3

## Figures and Tables

**Table 1:** Types of Behavior when the snails were given external stimulus.

Locality no.	Locality name	Bihevioral type (supplementary movie)			
		Defense	Offense	Others	In total
***Karaftohelix***(***Ainohelix***)***editha***					**21**
4	Wakkanai, Hokkaido, Japan	1 (Movie 1)			1
5-2	Rebun, Hokkaido, Japan	1			1
7	Nakatonbetsu, Hokkaido, Japan	2			2
13	Tomamae, Hokkaido, Japan	3			3
17	Kitami, Hokkaido, Japan	1			1
18	Furano, Hokkaido, Japan	1			1
19-1	Bibai, Hokkaido, Japan	1			1
22	Yubari, Hokkaido, Japan	3			3
25	Shimamaki, Hokkaido, Japan	2			2
28	Urakawa, Hokkaido, Japan	4			4
30-2	Samani, Hokkaido, Japan	2			2
***Karaftohelix***(***Ezohelix***)***gainesi***					**12**
7	Nakatonbetsu, Hokkaido, Japan		1		1
18	Furano, Hokkaido, Japan		1		1
19-1	Bibai, Hokkaido, Japan		2	1	3
24	Sapporo, Hokkaido, Japan		5 (Movie 2)		5
29	Okushiri, Hokkaido, Japan		1		1
31	Matsumae, Hokkaido, Japan		1		1
***Karaftohelix***(***Paraegista***)***takahidei***					**1**
20	Shakotan, Hokkaido, Japan	1			1
***Karaftohelix blakeana***					**6**
4	Wakkanai, Hokkaido, Japan	1 (Movie 3)		1	2
5-1	Funadomari, Rebun, Hokkaido, Japan	2		1	3
5-2	Rebun, Hokkaido, Japan	1			1
***Karaftohelix maackii***					**4**
2	Krasny Yar, Primorsky Krai, Russia	1			1
3	Russky, Primorsky Krai, Russia	3 (Movie 4)			3
***Karaftohelix middendorffi***					**4**
1	Bikin, Primorsky Krai, Russia	2			2
2	Krasny Yar, Primorsky Krai, Russia	2 (Movie 5)			2
***Karaftohelix selskii***					**4**
1	Bikin, Primorsky Krai, Russia		1 (Movie 6)	1	2
2	Krasny Yar, Primorsky Krai, Russia		1	1 (Movie 7)	2
***Karaftohelix ussuriensis***					**3**
2	Krasny Yar, Primorsky Krai, Russia	1			1
3	Russky, Primorsky Krai, Russia	2			2

**Table 2:** Summary of principal component analysis for the morphological analysis of shells.

Measurement	PC1	PC2
Eigenvalue	3.655	1.832
% of total variation	60.92	30.54
Coefficient		
H/D	0.581	0.808
AW/D	0.963	0.058
AH/D	0.948	0.158
SW/D	−0.882	0.359
SH/D	−0.137	0.974
NC/D	−0.833	0.271

## References

[b1] Darwin C. R. On the origin of species by means of natural selection, or the preservation of favoured races in the struggle for life . (John Murray, London, 1859). PMC5184128 30164232

[b2] Gittenberger E. What about non-adaptive radiation? Biol. J. Linn. Soc . 43, 263–272 (1991).

[b3] Gittenberger E. Radiation and adaptation, evolutionary biology and semantics. Org. Divers. Evol. 4, 135–136 (2004).

[b4] Schluter D. The ecology of adaptive radiation. Oxford Series in Ecology and Evolution . (Oxford University Press, New York, 2000).

[b5] Schluter D. Evidence for ecological speciation and its alternative. Science 323, 737–741 (2009). 19197053 10.1126/science.1160006

[b6] Nosil P. Ecological speciation. Oxford Series in Ecology and Evolution . (Oxford University Press, New York, 2012).

[b7] Schluter D. Ecology and the origin of species. TREE . 16, 372–380 (2001). 11403870 10.1016/s0169-5347(01)02198-x

[b8] Grant P. R. Ecology and evolution of Darwin’s finches . (Princeton University Press, New Jersey, 1986).

[b9] Meyer A. Phylogenetic relationships and evolutionary processes in East African cichlid fishes. TREE. 8, 279–284 (1993). 21236169 10.1016/0169-5347(93)90255-N

[b10] Losos J. B. . Contingency and determinism in replicated adaptive radiations of island Lizards. Science 279, 2115–2118 (1998). 9516114 10.1126/science.279.5359.2115

[b11] Sato A. . On the origin of Darwin’s Finches. Mol. Biol. Evol. 18, 299–311 (2001). 11230531 10.1093/oxfordjournals.molbev.a003806

[b12] Nosil P., Crespi B. J. & Sandoval C. P. Host-plant adaptation drives the parallel evolution of reproductive isolation. Nature . 417, 440–443 (2002). 12024213 10.1038/417440a

[b13] Gillespie R. Community assembly through adaptive radiation in Hawaiian spiders. Science . 303, 356–359 (2004). 14726588 10.1126/science.1091875

[b14] Clabaut C., Bunje P. M. E., Salzburger W. & Meyer A. Geometric morphometric analyses provide evidence for the adaptive character of the Tanganyikan cichlid fish radiations. Evolution . 61, 560–578 (2007). 17348920 10.1111/j.1558-5646.2007.00045.x

[b15] Gavrilets S. & Losos J. B. Adaptive radiation: contrasting theory with data. Science 323, 732–737 (2009). 19197052 10.1126/science.1157966

[b16] Losos J. B. Lizards in an evolutionary tree: Ecology and adaptive radiation of *Anoles*. (University of California Presss Ltd., London, 2009).

[b17] Rundell J. R. & Price D. Adaptive radiation, nonadaptive radiation, ecological speciation and nonecological speciation. TREE . 24, 394–399 (2009). 19409647 10.1016/j.tree.2009.02.007

[b18] Blankers T., Adams D. C. & Wiensa J. J. Ecological radiation with limited morphological diversification in salamanders. J. Evol. Biol . 25, 634–646 (2012). 22268991 10.1111/j.1420-9101.2012.02458.x

[b19] Simpson G. G. Tempo and mode in evolution . (Columbia University Press, New York, 1944).

[b20] Simpson G. G. The major features of evolution . (Columbia University Press, New York, 1953).

[b21] Lack D. Darwin’s finches . (Cambridge University Press, Cambridge, 1947).

[b22] Dobzhansky T. Genetics and the origin of species . (Columbia University Press, New York, 1951).

[b23] Futuyma D. J. Evolutionary Biology. (Sinauer Associates, Inc., Sunderland, MA, 1979).

[b24] Lima S. L. & Dill L. M. Behavioral decisions made under the risk of predation: a review and prospectus. Can. J. Zool. 68, 619–640 (1990).

[b25] Chesson P. & Kuang J. J. The interaction between predation and competition. Nature . 456, 235–238 (2008). 19005554 10.1038/nature07248

[b26] Hoso M. . A speciation gene for left–right reversal in snails results in anti-predator adaptation. Nat. Commun. 1, 133 (2010). 21139578 10.1038/ncomms1133 PMC3105295

[b27] Vermeij G. J. Evolution and Escalation. An Ecological History of Life. (Princeton University Press, Princeton, N. J, 1987).

[b28] Murray J., Clark B. & Johnson M. S. Adaptive radiation and community structure of Partula on Moorea. Proc. R. Soc. Lond. B Biol. Sci . 254, 205–211 (1993).

[b29] Chiba S. Accelerated evolution of land snails *Mandarina* in the oceanic Bonin Islands: Evidence from mitochondrial DNA sequences. Evolution 53, 460–471 (1999). 10.1111/j.1558-5646.1999.tb03781.x 28565404

[b30] Chiba S. Ecological and morphological patterns in communities of land snails of the genus *Mandarina* from the Bonin Islands. Biol. J. Linn. Soc . 17, 131–143 (2004). 10.1046/j.1420-9101.2004.00639.x 15000656

[b31] Davison A. Land snails as a model to understand the role of history and selection in the origins of biodiversity. Popul. Ecol. 44, 129–136 (2002).

[b32] Davison A. & Chiba S. Labile ecotypes accompany rapid cladogenesis in a land snail adaptive radiation. Biol. J. Linn. Soc . 88, 269–282 (2006).

[b33] Stankowski S. Extreme, continuous variation in an island snail: local diversification and association of shell form with the current environment. Biol. J. Linn. Soc . 104, 756–769 (2011).

[b34] Holland B. S. & Hadfield M. G. Origin and diversification of the endemic Hawaiian tree snails (Achatinellidae: Achatinellinae) based on molecular evidence. Mol. Phylogenet. Evol. 32, 588–600 (2004). 15223040 10.1016/j.ympev.2004.01.003

[b35] Cameron R. A. D. & Cook L. M. & Hallows J. D. Land snails on Porto Santo: adaptive and non-adaptive radiation. Philos. Trans. R. Soc. Lond. B. Biol. Sci . 351, 309–327 (1996).

[b36] Parmakelis A. . Inference of a radiation in *Mastus* (Gastropoda, Pulmonata, Enidae) on the island of Crete. Evolution . 59, 991–1005 (2005). 16136799

[b37] Sysoev A. & Schileyko A. Land snails and slugs of Russia and adjacent countries . Pensoft Publishers, Sofia, Moscow (2009).

[b38] Morii Y. . Evidence of introgressive hybridization between the morphologically divergent land snails *Ainohelix* and *Ezohelix*. Biol. J. Linn. Soc. 115, 77–95 (2015).

[b39] Imura Y. & Mizusawa K. The *Carabus* of the world. Shizawa Printing Co. Ltd ., Tokyo (in Japanese) (1996).

[b40] Barker G. M. Natural enemies of terrestrial molluscs . (CABI Publishing, London, 2004).

[b41] Konuma J. & Chiba S. Trade-offs between force and fit: extreme morphologies associated with feeding behavior in carabid beetles. Am. Nat. 170, 90–100 (2007). 17853994 10.1086/518182

[b42] Japan Wildlife Research Center. The national survey on the natural environment report of the distributional survey of Japanese animals (Land and fresh water mollusks). Biodiversity Center of Japan, Nature Conservation Bureau, Ministry of the Environment, Tokyo, Japan (2010, in Japanese).

[b43] Kawamichi M. Food, Food hoarding and seasonal changes of siverian chipmunks. Jpn. J. Ecol. 30, 211–220 (1980).

[b44] Azuma M. Colored illustrations of the land snails of Japan, Enlarged and revised edition, Hoikusha Publishing Co., Osaka (in Japanese) 1995.

[b45] Wade C. M., Mordan P. B. & Naggs F. Evolutionary relationships among the Pulmonate land snails and slugs (Pulmonata, Stylommatophora). Biol. J. Linn Soc . 87, 593–610 (2006).

[b46] Wade C. M. . Molecular phylogeny of the helicoid land snails (Pulmonata: Stylommatophora: Helicoidea), with special emphasis on the Camaenidae. J. Mollus. Stud. 73, 411–415 (2007).

[b47] Edgar R. C. MUSCLE: multiple sequence alignment with high accuracy and high throughput. Nucl. Acids Res . 32, 1792–1797 (2004). 15034147 10.1093/nar/gkh340 PMC390337

[b48] Castresana J. Selection of conserved blocks from multiple alignments for their use in phylogenetic analysis. Mol. Biol. Evol. 17, 540–552 (2000). 10742046 10.1093/oxfordjournals.molbev.a026334

[b49] Tanabe A. S. KAKUSAN: a computer program to automate the selection of a nucleotide substitution model and the configuration of a mixed model on multilocus data. Mol. Ecol. 7, 962–964 (2007).

[b50] Tanabe A. S. Kakusan4 and Aminosan: two programs for comparing nonpartitioned, proportional and separate models for combined molecular phylogenetic analyses of multilocus sequence data. Mol. Ecol. Resour . 11, 914–921 (2011). 21592310 10.1111/j.1755-0998.2011.03021.x

[b51] Ronquist F. & Huelsenbeck J. P. MrBayes 3: Bayesian phylogenetic inference under mixed models. Bioinformatics . 19, 1572–1574 (2003). 12912839 10.1093/bioinformatics/btg180

[b52] Rambaut A. & Drummond A. J. “TRACER version 1.5” Software distributed by the author at http://beast.bio.ed.ac.uk/Tracer (2007).

[b53] Jobb G. “TREEFINDER version of October 2008” Software distributed by the author at http://www.treefinder.de (2008).

[b54] Tamura K. . MEGA6: Molecular Evolutionary Genetics Analysis Version 6.0. Mol. Biol. Evol. 30, 2725–2729 (2013). 24132122 10.1093/molbev/mst197 PMC3840312

[b55] Felsenstein J. Confidence limits on phylogenies: An approach using the bootstrap. Evolution . 39, 783–791 (1985). 10.1111/j.1558-5646.1985.tb00420.x 28561359

[b56] Ohba N. The mystery of fireflies. Yokosuka City Museum,Yokosuka (2004, in Japanese).

[b57] Konuma J., Nagata N. & Sota T. Factors determining the direction of ecological specialization in snail-feeding carabid beetles. Evolution 65, 408–418 (2011). 20977474 10.1111/j.1558-5646.2010.01150.x

[b58] Konuma J., Sota T. & Chiba S. A maladaptive intermediate form: a strong trade-off revealed by hybrids between two forms of a snail-feeding beetle. Ecology 94, 2638–2644 (2013). 24400515 10.1890/12-2041.1

[b59] Tattersfield P. Variation in foot size and shape in some British land snails and its functional significance. Biol. J. Linn. Soc . 36, 365–376 (1989).

[b60] Wainwright P. C. & Reilly S. M. Ecological morphology: integrative organismal biology . (University of Chicago Press, Chicago, 1994).

[b61] Kimura K., Hirano T. & Chiba S. Assortative mating with respect to size in the simultaneously hermaphroditic land snail *Bradybaena pellucida*. Acta Ethol . 18, 265–268 (2015).

[b62] Hayashi M. & Chiba S. Intraspecific diversity of mitochondria1 DNA in the land snail *Euhadra peliomphala* (Bradybaenidae). Biol. J. Linn. Soc . 70, 391–401 (2000).

[b63] Brown J. S. & Vincent T. L. Organization of predator-prey communities as an evolutionary game. Evolution . 46, 1269–1283 (1992). 10.1111/j.1558-5646.1992.tb01123.x 28569003

[b64] Rintelen T. V., Wilson A. B., Meyer A. & Glaubrecht M. Escalation and trophic specialization drive adaptive radiation of freshwater gastropods in ancient lakes on Sulawesi, Indonesia. Proc. R. Soc. Lond. B Biol. Sci . 271, 2541–2549 (2004). 10.1098/rspb.2004.2842 PMC1691893 15615679

[b65] Schilthuizen M. . Microgeographic evolution of snail shell shape and predator behavior. Evolution 60, 1851–1858 (2006). 17089969

