# Supplementary material for: Parallel evolution of passive and active defence in land snails
Source: Sci Rep. 2016 Nov 11;6:35600. doi: 10.1038/srep35600 (PMC5105203; doi:10.1038/srep35600)
Supplement: Supplementary Information [file srep35600-s1.pdf]

TITLE

Parallel evolution of passive and active defence in land snails

AUTHOR

Yuta Morii 1,2, Larisa Prozorova 3, & Satoshi Chiba 1

1 Division of Regional Ecosystem Studies, Department of Environmental Life Sciences, Graduate School of Life Sciences, Tohoku University, Sendai 9808576, Japan. 2 Division of Environmental Resources, Research Faculty of Agriculture, Hokkaido University, Sapporo 0608589, Japan. 3 Institute of Biology and Soil Science, Far East Branch, Russian Academy of Sciences, Vladivostok 690022, Russia.

Supplementary Informations

Supplementary Movie 1 | The passive defence behaviour of *Karaftohelix editha*.

Supplementary Movie 2 | The active defence behaviour of *Karaftohelix gainesi*.

Supplementary Movie 3 | The passive defence behaviour of *Karaftohelix blakeana*.

Supplementary Movie 4 | The passive defence behaviour of *Karaftohelix maackii*.

Supplementary Movie 5 | The passive defence behaviour of *Karaftohelix middendorffi*.

Supplementary Movie 6 | The active defence behaviour of *Karaftohelix selskii*.

Supplementary Movie 7 | The creating bubbles behaviour of *Karaftohelix selskii*.

Supplementary Movie 8 | The passive defence behaviour of *Karaftohelix editha* against *Acoptolabrus gehinii*.

Supplementary Movie 9 | The active defence behaviour of *Karaftohelix gainesi* against *Acoptolabrus gehinii*.

Supplementary Movie 10 | The active defence behaviour of *Karaftohelix gainesi* against *Acoptolabrus gehinii*.
